# Editorial: Visual Language

**DOI:** 10.3389/fpsyg.2019.01765

**Published:** 2019-08-02

**Authors:** Wendy Sandler, Marianne Gullberg, Carol Padden

**Affiliations:** ^1^Sign Language Research Lab, University of Haifa, Haifa, Israel; ^2^Centre for Languages and Literature, Lund University, Lund, Sweden; ^3^Department of Communication and Center for Research in Language, University of California San Diego, San Diego, CA, United States

**Keywords:** sign language, gesture studies, multimodality, iconicity, visual language

Traditionally, research on human language has taken speech and written language as the main domains of investigation. Visual aspects of language have therefore long been excluded from study. However, the advent of technology allowing us to capture and include auditory and visual signals in the study of language has changed the landscape. There is now a wealth of empirical studies documenting visual aspects of language, ranging from rich studies of sign languages, the most highly sophisticated and self-contained visual language systems, to the burgeoning field of gesture studies, which targets speech-associated gestures, facial expressions, and other bodily movements related to communicative expressions as new domains of study.

However, despite the large body of work now available documenting visual elements of language, sign language and gesture are rarely treated together in theoretical discussions of the human language faculty. Sign language studies often search for linguistic structures that are derived from spoken language theory. Conversely, gesture researchers refrain from defining gestures as “linguistic” (although they often insist that they are part of “language”), because they do not conform to certain properties that linguists consider defining properties, such as strict compositional structure and syntactic rules. In both cases, definitions and concomitant exclusions are not necessarily enlightening, since both domains—speech-associated gestures and sign language—naturally exploit visual expression, and must both be considered in attempting to arrive at a comprehensive account of the human language faculty. By considering both types of visual language, the 19 papers in this *Frontiers Research Topic* volume thus transcend theoretical—and, we would say, artificial—divides. The collection aims to pave the way for an inherently multimodal view of language, in which visible actions of the body play a crucial role.

The volume treats four broad topics: (1) the multimodal nature of language; (2) multi modal representation of meaning; (3) multimodal and multi channel prosody; and (4) acquisition and development of visual language in children and adults. This division aims to organize the Research Topic for the reader, although there is some inevitable overlap.

The first topic targets the nature of all language as multimodal, examining the relationships between speech, gestures, and sign. Visible parts of the body can be engaged in language use in a range of ways, and the papers in this section illustrate specific language phenomena that are multimodal. Perniss; Ferrara and Hodge both review evidence to support a multimodal model of language that accounts for how humans coordinate their semiotic repertoires in crossmodal and composite ways. These authors draw on fundamental modes of communication, including depiction, description, and indicating (Clark, 1996, 2016). Both papers also stress the need to consider the wider context in which utterances are constructed and interpreted, in order to fully understand how multimodal resources are integrated into language as traditionally defined. Müller delves deep into the theoretical debates concerning the status of gestures relative to speech, and addresses the question—are gestures part of language or *are* they language themselves? She further discusses the relationship between the speech-gesture ensemble and sign language, specifically targeting the issue of whether the systems are fundamentally different in nature, or whether there is a continuum. Sandler argues for the centrality of the body in understanding the nature of a central property of language: compositionality. She details the linguistic functions of different bodily articulations in the prosodic, lexical, and pragmatic structure of established sign languages, and their recruitment in the emergence of new sign languages, illuminating more general principles of compositionality common to spoken and visual languages alike. The paper goes on to seek possible evolutionary roots of communicative compositionality in physical displays of intense emotions by athletes, and their interpretation. Dachkovsky et al. focus on the relationship between linguistic complexity and its expression by the body, in the emergence of a young sign language, Israeli Sign Language. Drawing on narratives produced by three generations of signers, the authors illustrate how the self-organization of bodily articulations becomes more systematic and reduced as the language becomes more complex over time. Finally, Liebal and Oña discuss the search for the roots of human language in a cross-species comparative approach, and investigate whether precursors to language may already be present in our closest relatives, the non-human primates. They review the debate concerning whether non-human primates use gestures to “mean” the same as humans, and present an overview of how different approaches to visual/gestural vs. vocal communication in non-human primates lead to different answers.

While the first topic deals with different kinds of structure conveyed in language, the second broad topic concerns how meaning can be represented multimodally, and the ways in which meaningful elements can be quantified and modeled. The papers in this section address issues such as how the body, and specifically the hands, can create meaning visually and kinetically in speech-associated gestures and sign languages. Mittelberg begins with a discussion of meaning-making in speech-associated gestures which involves iconicity (a direct form-meaning correspondence), indexicality (contiguity), and habit (conventionality). Comparing two ways in which meaning can be extended in language, metonymy and metaphor, she argues that metonomy is a more basic principle in gestures and signs than metaphor. Mittelberg describes metonymy as more experientially grounded than metaphor, as it highlights a partial aspect of a larger context of human activity, the activity itself being expressed within a *frame*, or a context of experience. Metonymic gestures are simultaneously indexical and refer to conventions of human practice. Cooperrider et al. explore a single gestural form, the so-called epistemic palm up, as a starting point for examining a network of meanings that appear to be similar across gesture and sign. These comparisons serve as the basis for a discussion of the origins of communicative forms, how they divide into multiple different meanings, and become integrated into language. In an unusual comparative study across language modalities, Perlman et al. examine the presence of iconicity in two signed languages (American Sign Language and British Sign Language) and two spoken languages (Spanish and English). The analyses reveal characteristic patterns of iconicity across semantic domains both within and across the languages depending on the affordances of the main modality. Three further papers focus on iconicity in sign languages specifically: Lu and Goldin-Meadow examine depiction in American Sign Language to reveal a conventional (more lexicalized) and, at the same time, a so-called embellished (more gesture-like) kind of depiction, explaining that the preference depends on context and task. Meir and Cohen investigate metaphors in Israeli Sign Language. They provide a detailed analysis of the ways in which metaphors in sign language differ from metaphors in spoken language, and suggest two principles to account for these differences. They conclude that all human languages exploit metaphorical expression to convey vivid sensory images, while the visual and the auditory modalities impose different constraints on such expression. The fact that the body is visible while signing determines the ways in which signers can refer metaphorically to the body for both human and non-human properties. Finally, in a methodologically oriented paper, Östling et al. use computer based tools to automatically process 120,000 videos from 31 sign languages to reveal two different cross-linguistic patterns of iconicity: the use of two hands to represent plurality, and of locations on different parts of the body to represent activities associated with such locations (e.g., the head with thinking). Computational modeling is a revealing tool for simulating natural communication and testing its interpretation. Ravenet et al. describe the challenges involved in modeling multimodal behavior for so-called Embodied Conversational Agents (ECAs). They identify elements that need to be captured regarding speech and gesture in order to automatically generate multimodal communicative behavior in successful virtual/robotic conversational partners.

The third topic in the volume is concerned with prosody that is multimodal (speech and gesture) and multi channel (manual and non-manual in sign language). Prosody refers to linguistic cues such as intonation, tone, stress, and rhythm, which are superimposed on the morphosyntactic language stream. Both in the domain of sign language and in gesture studies, empirical studies of the coordination of visual prosodic cues with the phrases and sentences of language are quite rare (but for pioneering work, see e.g., Nespor and Sandler, 1999; Sandler, 2010 and Sandler this volume for sign language; McClave, 1994 for gesture). Shattuck-Hufnagel and Ren examine the precise nature of the temporal relationship between speech and one type of co-speech gesture in adults, looking at how non-referential gestures in academic lectures coordinate with prosodic prominence in speech. The analyses reveal a tight link between the prosodic structure of spoken utterances and bodily movements, supporting the claim that a comprehensive speech production model must generate and align gesture and speech as part of the same system. Esteve-Gibbert and Guellaï contextualize and evaluate a range of studies on the development of the prosodic coordination of speech and gesture in childhood. Brentari et al. focus on visible prosodic markers in the manual and non-manual channels of different types of imperatives in American Sign Language (ASL). They also test their comprehension by signers of ASL, as well as by signers of a different sign language (German Sign Language, DGS), and finally by hearing non-signers. Results show that different speech acts display different patterns, and also, importantly, that the patterns are sign language-specific.

The fourth topic deals with acquisition and development of visual language, both in child and adult language learners considering both sign language and gestures. Janke and Marshall ask whether speech-associated gestures function as a useful starting point or scaffold for hearing adults learning sign language, and whether iconic signs are easier to learn than less iconic signs, as is often claimed. The results suggest that adult hearing learners cannot straightforwardly draw on gestures, whether iconic or not. Instead, the challenge seems to be to reduce gestural resources and “linguisticize” a small number of hand shapes to arrive at forms that are part of the grammar of a sign language. Shield and Meier also examine children's and adults' acquisition of sign language and posit four possible strategies for learning signs/imitating gestures. They review evidence from typical and atypical hearing and deaf groups to reveal different developmental trajectories across typical and atypical populations. Finally, Graziano and Gullberg focus on the well-rooted assumption that gesture is mainly a compensatory device to support speaking difficulties. Analyses of fluent and disfluent speech from both adult competent speakers of different languages and child and adult language learners instead suggest that gestures are integrated with speech such that both modalities are affected by speech production difficulties. The results thus support an integrated view of speech and gesture and of a view of language use as fundamentally multimodal.

In conclusion, the papers in this volume provide new evidence for the role of visual elements expressed by the body in language. The volume unifies theoretical and empirical proposals toward a more comprehensive view of the multimodal nature of language, in which speech, gestures, and sign are treated on a par. We hope that the volume will provide additional substance to Perniss' conclusion that “[W]e are already on the threshold of a new paradigm.”

## Author Contributions

All authors listed have made a substantial, direct and intellectual contribution to the work, and approved it for publication.

### Conflict of Interest Statement

The authors declare that the research was conducted in the absence of any commercial or financial relationships that could be construed as a potential conflict of interest.
